# Expression of Phenotypic Astrocyte Marker Is Increased in a Transgenic Mouse Model of Alzheimer's Disease versus Age-Matched Controls: A Presymptomatic Stage Study

**DOI:** 10.1155/2016/5696241

**Published:** 2016-09-08

**Authors:** Aurélie Doméné, Chelsea Cavanagh, Guylène Page, Sylvie Bodard, Christophe Klein, Cécile Delarasse, Sylvie Chalon, Slavica Krantic

**Affiliations:** ^1^UMR Inserm U930, Université François Rabelais, Tours, France; ^2^Department of Neuroscience, Douglas Hospital Research Centre, Montreal, QC, Canada H4H 1R3; ^3^EA 3808 CiMoTheMA, Université de Poitiers, Poitiers, France; ^4^Centre de Recherche des Cordeliers, INSERM, Université Paris Descartes, Sorbonne Paris Cité, UMR_S 1138, Université Pierre et Marie Curie Université Paris 06, Sorbonne Universités, Paris, France; ^5^Institut du Cerveau et de la Moelle épinière (ICM), CNRS UMR 7225, INSERM U 1127, UPMC-P6 UMR S 1127, Paris, France

## Abstract

Recent mouse studies of the presymptomatic stage of Alzheimer's disease (AD) have suggested that proinflammatory changes, such as glial activation and cytokine induction, may occur already at this early stage through unknown mechanisms. Because TNF*α* contributes to increased A*β* production from the A*β* precursor protein (APP), we assessed a putative correlation between APP/A*β* and TNF*α* during the presymptomatic stage as well as early astrocyte activation in the hippocampus of 3-month-old APPswe/PS1dE9 mice. While Western blots revealed significant APP expression, A*β* was not detectable by Western blot or ELISA attesting that 3-month-old, APPswe/PS1dE9 mice are at a presymptomatic stage of AD-like pathology. Western blots were also used to show increased GFAP expression in transgenic mice that positively correlated with both TNF*α* and APP, which were also mutually correlated. Subregional immunohistochemical quantification of phenotypic (GFAP) and functional (TSPO) markers of astrocyte activation indicated a selective and significant increase in GFAP-immunoreactive (IR) cells in the dentate gyrus of APPswe/PS1dE9 mice. Our data suggest that subtle morphological and phenotypic alterations, compatible with the engagement of astrocyte along the activation pathway, occur in the hippocampus already at the presymptomatic stage of AD.

## 1. Background

Alzheimer's disease (AD) is an age-related, incurable neurodegenerative disorder [1]. The clinical symptoms of AD include loss of cognitive functions that interfere with the individuals' ability to perform daily tasks, trouble remembering recent events, and eventually total memory loss, in addition to a host of other symptoms such as agitation, paranoia, sleep disturbances, aggression, and motor dysfunctions [2]. AD is characterized histologically by extracellular deposits of amyloid beta (A*β*) into plaques and intracellular accumulation of hyperphosphorylated tau protein (neurofibrillary tangles). However, AD pathogenesis is still incompletely elucidated. The prevailing theory on the cause of AD is the amyloid cascade hypothesis, which posits that the overproduction of A*β* from the amyloid precursor protein (APP) initiates a series of events, including synaptic dysfunction, hyperphosphorylation of tau, and neuroinflammation-related glia activation, which culminates in widespread neuronal death [1].

Neuroinflammation is associated with a heavy A*β* plaque burden and accumulation of neurofibrillary tangles. Various GWAS studies have implicated a number of genes related to the immune system in the sporadic form of AD, including* CR1*,* CD33*, and* CLU* [3]. Moreover,* CLU* is an acute phase protein and therefore is a marker of an increased inflammatory response [4]. In particular, genomic studies have further revealed a significant association between TNF*α* polymorphisms and AD [5] and TNF signaling has been associated with conversion to dementia in patients with mild cognitive impairment (MCI) [6].

A growing body of data supports a role for cytokines and other inflammatory mediators in neuronal activities, including learning, memory, and neural plasticity [7]. Although TNF*α* is a well-known immune mediator, according to an emerging concept, TNF*α* is also an important regulator of synaptic function and excitability [8–10]. Since synaptic dysfunction is ultimately responsible for cognitive impairments in AD, the effect TNF*α* has on synaptic integrity is crucial to understanding disease pathogenesis. Moreover, immune mediators, including TNF*α*, are not limited to classical immune functions and there is significant crossover between systems. For example, the mitochondrial translocator protein (TSPO), whose ligands are currently used as markers of reactive gliosis and brain inflammation in AD patients with overt pathology [11], was initially characterized for its role in steroid synthesis by translocating cholesterol from the outer to the inner mitochondrial membrane [12]. TSPO is indeed likely more than a marker of inflammation since intervention and treatment with TSPO ligands demonstrated reduced neuropathology and behavioral impairments in a mouse model of AD, at least at the advanced stage of the disease [13]. However, there is currently no consensus about the precise type of glial cells that respond to a proinflammatory insult via TSPO upregulation. Some studies have reported the increase of TSPO expression in both microglia and astrocytes [14, 15], whereas other studies pointed to a selective microglia expression [16].

Recent functional Magnetic Resonance Imaging (fMRI) studies have pointed to hyperactivation of hippocampal neuronal networks and functionally related cortical areas in patients with mild cognitive impairment [17, 18], which may be considered as a prodromal stage of AD. In addition to hyperactivation in fMRI studies, there is a clear association between epileptiform activity and AD such that seizure prevalence is increased in sporadic as well as in familial AD cases [19]. Alterations in network activity due to increased seizures are also apparent in various mouse models of AD. For example, preplaque (presymptomatic) TgCRND8 mice display increased susceptibility to pentylenetetrazole-induced seizures in comparison to control mice [20]. Accordingly, APPswe/PS1dE9 mice have an increased incidence of unprovoked seizures at the onset of A*β* deposition [21]. Further evidence suggests that the accumulation of soluble A*β* leads to spontaneous, nonconvulsive epileptiform activity, compensatory remodeling of inhibitory GABAergic neurotransmission, and deficits in synaptic plasticity [22].

We have recently reported that signs of microglial engagement into the activation process coincide with increased TNF*α* expression in the hippocampus of a preplaque TgCRND8 mouse model of AD [23]. Moreover, these early alterations appear to be concomitant with the altered synchronization of hippocampal neuronal network activities [24], in line with the current view on the role of TNF*α* in the regulation of neuronal activity [25]. The aim of the present study was to assess whether similar early alterations in glia activity during the presymptomatic, preplaque stage may contribute to the previously reported hyperexcitability of hippocampal neurons in another mouse AD model, APPswe/PS1dE9 [21]. We used APPswe/PS1dE9 mice of the same age (3-month-old) as in the previous study [21] to specifically address the involvement of astrocytes in the control of synaptic function based on the fact that glial cells, together with presynaptic and postsynaptic neurons, are an intrinsic part of the “tripartite” synapse [26]. To do so, we compared the levels of hippocampal TNF*α* with the expression of phenotypic (GFAP) and functional (TSPO) astrocyte markers.

## 2. Materials and Methods

### 2.1. Chemicals and Antibodies

Sodium fluoride (NaF), phenylmethylsulfonyl (PMSF), protease and phosphatase inhibitor cocktails, ethylenediamine triacetic acid (EDTA), paraformaldehyde (PFA), Triton X-100, and 4′,6-diamidino-2-phenylindole (DAPI) were purchased from Sigma (Saint-Quentin-Fallavier, France). Normal Horse Serum (NHS) and fluorescent mounting medium (Fluoromount) were from Dako (Les Ulis, France).

Anti-glial fibrillary acidic protein (GFAP) antibody was purchased from Synaptic systems (ab173004, Göttingen, Germany) and anti-TSPO 18 kDa from Novus Biologicals (NBP1-95674, Interchim distributor, Montluçon, France). Donkey anti-Guinea pig conjugated with Alexa Fluor 647 and donkey anti-rabbit conjugated with Alexa Fluor 546 were purchased from Cell Signaling (Millipore, Fontenay-sous-Bois, France). For Western blot, the following additional polyclonal primary antibodies were used: anti-GFAP (Z0334; Dako, Les Ulis, France), anti-TNF*α* (AB2148P; Chemicon, Millipore, Fontenay-sous-Bois, France), and CT20-anti APP antibody (171610; Chemicon, Millipore, Fontenay-sous-Bois, France). The following are secondary antibodies conjugated with Horseradish Peroxidase (HRP): goat-anti rabbit IgG-HRP (sc-2004; Santa Cruz Biotechnology, CliniSciences, Nanterre, France) and horse-anti goat IgG-HRP (Vector PI-9500, Malakoff, France). Anti *β* (I-19)-actin-HRP (sc1616-HRP) was also purchased from Santa Cruz Biotechnology, CliniSciences, Nanterre, France). All other antibodies used in Western blot experiments were the same as those used for immunohistochemistry.

### 2.2. Animals

Double APPswe/PS1dE9 transgenic and WT mice at the age of 3 months were used in this study. These transgenic mice express chimeric mouse/human APP DNA sequence containing the K595N/M596L Swedish mutation and human PS1 variant carrying the exon 9 deletion driven by hamster prion promoter elements directing the expression to central nervous system neurons [27].

To create the mouse colony, male hemizygote B6C3-Tg (APPswe,PS1dE9)85Dbo (stock #004462) and female wild type mice (B6C3F1, stock #10010) obtained from Jackson Laboratories (Bar Harbor, ME USA) were bred under standard conditions. The mice were individually housed under standard enriched environment in a room with a 12h/12h light/dark cycle (lights on from 7:00 to 19:00) under steady temperature (21 ± 1°C) and humidity (55 ± 5%) with access to food and water ad libitum. Only female mice were included in this study since it is generally recognized that, in both mice and humans, females are at a higher risk of developing AD [28], a trait that may be relevant for identification of the earliest alterations at the studied presymptomatic stage of AD.

Animals were treated in accordance with the European Community Council Directive 2010/63/EU for laboratory animal care and the experimental protocol was validated by the Regional Ethical Committee (Authorization number: 2013-01-23).

### 2.3. Tissue Processing

The experiments performed in this study were run on two independent cohorts of mice. A total of *n* = 4 WT and *n* = 5 APPswe/PS1dE9 mice were used in the first cohort, whereas the second cohort included *n* = 5 WT and *n* = 6 APPswe/PS1dE9 mice. Mice from the first cohort were used for in situ experiments (autoradiography and immunofluorescence), whereas the mice from the second cohort were used for both in situ (autoradiography) and biochemical (Western blot and ELISA) analysis.

The mice were sacrificed by decapitation under light isoflurane anesthesia, and their brains were carefully removed on ice. For in situ studies, the whole brain or the right hemisphere was immediately frozen in isopentane cooled at −35°C in the first and second experimental cohorts, respectively. Brains were sliced sagittally using a freezing cryostat (CM 3050S, Leica, Germany) at 16 *µ*m and mounted on gelatinized Super-Frost slides (CML, Nemours, France). Sections were stored at −80°C for at least 4 days until use. After pooling data from both cohorts, the total number of mice assessed for autoradiography was *n* = 9 WT and *n* = 11 APPswe/PS1dE9.

Immunohistochemical experiments were performed on the sections adjacent to those used for autoradiography and they were all from the first experimental cohort (*n* = 4 WT and *n* = 5 APPswe/PS1dE9). However, the adjacent sections from the WT mouse ID 672 were partially damaged and could not be processed. Consequently, the WT mouse ID 672 was withdrawn and the total number of mice assessed by immunofluorescence and used for the statistical analysis was therefore *n* = 3 for WT and *n* = 5 for APPswe/PS1dE9.

Biochemical experiments were performed exclusively on the mice sacrificed in the second experimental cohort. Hippocampi were extracted from the left brain hemispheres coming from a total of *n* = 5 WT and *n* = 6 APPswe/PS1dE9 mice. They were immediately homogenized in 10 volumes of lysis buffer (25 mmol/L Tris-HCl, 150 mmol/L NaCl, and 1 mmol/L EDTA, pH 7.5) supplemented with 50 mmol/L NaF, 1 mmol/L PMSF, and protease and phosphatase inhibitor cocktails (50 *μ*L/g of tissue and 10 *μ*L/mL of lysis buffer, resp.). Lysates were sonicated and centrifuged at 15,000 ×g for 15 minutes at 4°C. The resulting supernatants (dilution 1 : 4) were collected to measure the quantity of total protein with the Bradford method. Samples were aliquoted and stored at −80°C until Western blot analysis and ELISA assay as described in Sections 2.6 and 2.7.

### 2.4. Autoradiographic Study of TSPO Binding Sites

The autoradiographic study was conducted on the entire cohort of animals used in this study coming from both experimental cohorts (a total of *n* = 9 WT and *n* = 11 APPswe/PS1dE9 mice). To scan the hippocampus region, 4 sets of slides were treated. Per set, 2 slides were used to determine the total binding and 1 slide was used for the nonspecific binding. A total of 48 (4 sets of slides × 3 slides per set × 4 sections per slide) sagittal sections per mouse were used for each mouse.

The density of TSPO binding sites was measured by in vitro autoradiography using [^3^H]PK-11195 (Specific Activity 3.06 GBq/*μ*mol, Perkin-Elmer) at 1 nmol/L in 50 mmol/L Tris-HCl buffer, pH 7.4. Brain sections were allowed to equilibrate at room temperature (RT) for 3 hours and then incubated with 1 nmol/L [^3^H]PK-11195 in 50 mmol/L Tris-HCl buffer, pH 7.4, at RT for 60 minutes. Nonspecific binding was assessed in the presence of 1 *μ*mol/L PK-11195 (Sigma-Aldrich, Les Abresles, France). Slides were rinsed twice in ice cold buffer (4°C) for 5 minutes and then briefly in distilled water at 4°C and dried at RT. Dry slides were made conductive by an application of metal electric tape (3 M, Euromedex, Strasbourg, France) on the free side and then placed in the gas chamber of the *β*-imagerTM 2000 (Biospace Lab, Paris, France). Data from brain sections were collected during 2 hours. In agreement with the cresyl violet staining (data not shown) and the Allen Brain atlas (http://mouse.brain-map.org/static/atlas; from lateral 1.725 mm), sagittal hippocampus sections were selected manually. Using the *β*-vision software (Biospace Lab, Paris, France), the level of bound radioactivity was directly determined by counting the number of *β*-particles emitted from the delineated area. The radioligand signal in the region of interest was measured on at least eight sections for each mouse and expressed as counts per minute per square millimeter (cpm/mm^2^). Specific binding was determined by subtracting nonspecific binding from total binding.

### 2.5. Immunofluorescence Study of TSPO Expression: Colocalization with GFAP

Eight sagittal sections (16 *µ*m thick) per mouse were used for immunofluorescence experiments. These 8 sections were adjacent to those in the 2 slides used to determine the total binding in autoradiography experiments from one (out of 4 used for autoradiography) set of slides, which was anatomically matched for all mice. A total of 8 (*n* = 3 WT and *n* = 5 APPswe/PS1dE9) mice, all coming from the first experimental cohort, were used.

After 10 minutes at RT, sections were delineated using the pencil Dakopen on the glass slide and fixed in PFA 4% at RT for 30 minutes. Sections were washed 3 times in PBS 0.1 mol/L for 5 minutes at RT and tissue sections were incubated for 2 hours at RT in a buffer to enhance cell permeability and to block nonspecific sites (PBS 0.1 mol/L/0.3% Triton X-100/5% NHS) before incubation overnight at 4°C with both 1 : 500 diluted polyclonal Guinea pig anti-GFAP (173 004; Synaptic Systems, Göttingen, Germany) and 1 : 1000 diluted monoclonal rabbit anti-TSPO (clone EPR5384, NBP1-95674; Novus Biologicals, Abingdon, United Kingdom). Primary antibodies were diluted in PBS 0.1 mol/L/0.3% Triton X-100/1% NHS. After 4 washings with PBS 0.1 mol/L at RT for 5 minutes, sagittal sections were incubated 2 hours at RT in the dark with secondary antibodies, each at a 1 : 400 dilution in PBS 0.1 mol/L/Triton X-100/1% NHS: either donkey anti-Guinea pig Alexa Fluor 647 or donkey anti-rabbit Alexa Fluor 546. The slices were washed 5 times for 5 minutes in PBS 0.1 mol/L and twice in distilled water and incubated with 1 : 10 000 diluted DAPI for 15 minutes at RT. After 3 washings in distilled water, the slices were mounted with fluorescent mounting medium and kept in a dark box at 4°C until observations and analysis.

Images from immunolabeled hippocampal sections were captured with AxioVision Rel 4.8 software (Carl Zeiss, Oberkochen, Germany) using a fluorescence laser scanning microscope with a 20x objective (Olympus BX51, Rungis, France). Images of total hippocampus were obtained using MosaiX module and multiple fluorescence signals were acquired sequentially to avoid crosstalk between image channels. The images were merged and then analyzed by using ImageJ software (win 32, Rasband, WS, ImageJ, US National Institute of Health, Bethesda, MD, USA). Hippocampus regions were determined as per Franklin and Paxinos atlas (2007): CA1 and CA2 were combined (CA1-2) due to the relatively amorphous boundary separating these two regions. CA3 and dentate gyrus (DG) were also analyzed.

A cell was considered as positive only when its nucleus was detectable (DAPI positive). For each subregion of the hippocampus, the number of GFAP-immunoreactive (IR) and double GFAP/TSPO-IR cells was, respectively, counted by ImageJ with the plugin cell counter. For each subregion of the hippocampus (CA1-2, CA3, and DG), the total cell count per square millimeter was calculated from 4 sections per mouse in each group and then averaged to obtain a mean cell count per unit area.

### 2.6. Western Blot Studies

Samples (20 *µ*g proteins of hippocampus lysates) coming exclusively from the second experimental cohort and corresponding to *n* = 5 WT and *n* = 6 APPswe/PS1dE9 mice were processed for electrophoresis by adding Laemmli sample buffer and denatured by boiling for 5 minutes at 100°C. The proteins were then separated by SDS-PAGE, on a Tris-Glycine 4–20% gradient gel (Invitrogen, Fisher Scientific distributor, Illkirch, France), at 130 V for 2 hours. Proteins were transferred to nitrocellulose membrane (0.45 *µ*m; Amersham, GE Healthcare, Life Sciences, Vélizy-Villacoublay, France) at 80 V for 35 minutes at 4°C, under agitation. Blots were blocked for 1 hour at RT with 5% skim milk diluted in Tris-buffered saline (TBS) containing 0.05% Tween (TBS-T) and then incubated with primary antibodies overnight at 4°C in 1% skin milk diluted in TBS-T. The antibodies used were CT20 (1 : 5000) or 6E10 (1 : 1000) anti-APP recognizing both human and murine (CT20) or exclusively human (6E10) immunopositive protein of 112 kDa, anti-TSPO (1 : 3000) detecting a 15 kDa protein, anti-TNF*α* (1 : 1000) which detects a 51 kDa immunoreactive protein, and anti-GFAP (1 : 5000) which specifically detects a 55 kDa protein.

After 3 washes of 5 minutes in TBS-T, membranes were incubated with secondary antibodies for 1 hour at RT in 1% milk TBS-T. The corresponding HRP-conjugated anti-IgG was applied as a secondary antibody (1 : 5000) in parallel with the HRP-conjugated anti-*β*-actin IgG (1 : 2000), used as an internal standard for equal loading.

Immunoreactive proteins were revealed using Western lightning chemiluminescence Reagent Plus kit (Perkin-Elmer, Whatman, MA, USA) followed by densitometric analysis with ImageJ software (win 32, Rasband, WS, ImageJ, US National Institute of Health, Bethesda, MD, USA). The optical density (OD) measured for each immunoreactive band was normalized to *β*-actin. The results were expressed as relative optical density (ROD).

### 2.7. ELISA

A commercially available ELISA kit was used for TNF*α* (sensitivity 1 pg/mL) quantification according to the manufacturer's instructions (Biosensis, Thebarton, Australia). The range of analysis was between 15.6 and 1000 pg/mL. Homogenates from hippocampi (10 mg of tissue/100 *µ*L) coming from the second experimental cohort and corresponding to *n* = 5 WT and *n* = 6 APPswe/PS1dE9 mice were added to precoated wells and incubated at RT. The enzymatic reaction was stopped after 5–20-minute incubation in the dark with tetramethylbenzidine substrate and the OD was read at 450 nm within 30 minutes, using the Benchmark Plus spectrum spectrophotometer (Bio-Rad). The cytokine level was then calculated by plotting the OD of each sample against the standard curve.

Human A*β* (1–42) ELISA (sensitivity < 10 pg/mL; concentration range: 15.6 to 1000 pg/mL) was used to quantify the A*β* expression in hippocampal homogenates as per manufacturer's (Thermo Fischer Scientific, Saint Aubin, France) instructions.

### 2.8. Statistical Analysis

GraphPad Prism 5 (GraphPad Software, San Diego, CA) was used for all statistical analyses except effect sizes, which were calculated using the Colorado Springs Online Effect Size Calculator, University of Colorado (http://www.uccs.edu/~lbecker/) for Cohen's *d* and G^*∗*^ Power for *r*. Cohen's *d* and *r* were calculated to estimate the power to detect the statistically significant differences between groups and correlations, respectively. The effect size was evaluated according to the following convention: *d* = 0.2 for small, *d* = 0.5 for medium, and *d* = 0.8 for large and *r* = 0.1 for small, *r* = 0.3 for medium, and *r* = 0.5 for large. The results are expressed as means ± standard error of the mean (SEM). After normality was confirmed using the Shapiro-Wilk test of normality, two-tailed, unpaired Student's *t*-tests were used to compare the 2 groups of mice (WT versus APPswe/PS1dE9). The correlative analysis of biochemical data was performed by linear regression. In all cases, significance was noted at *p* < 0.05 (*∗*) and *p* < 0.01 (*∗∗*). Figure montages were created using free image editing software, the GNU Image Manipulation Program (GIMP).

## 3. Results

### 3.1. Hippocampal APP and TNF*α* Expression Indicates a Presymptomatic Stage of AD-Like Pathology in 3-Month-Old APPswe/PS1dE9 Mice

**Figure 1:**
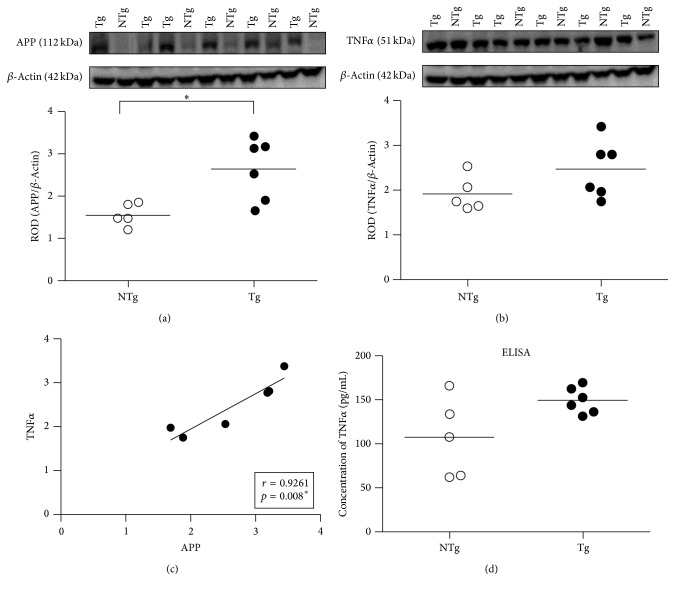
APP and TNF*α* expression in the hippocampus of 3-month-old APPswe/PS1dE9 mice. (a) APP (112 kDa) and (b) TNF*α* (51 kDa) expressions were determined by Western blotting. Equal protein loading was ascertained by the parallel assessment of *β*-actin (42 kDa) and results were expressed as the relative optical density (ROD). (c) In APPswe/PS1dE9 mouse group, the increase in APP correlates positively with TNF*α* (*r* = 0.9261; *p* = 0.0080). Correlation analysis between respective ROD values as determined in Western blot studies (a and b) was performed by linear regression. (d) The level of TNF*α* was also assayed by ELISA and expressed in pg/mL protein. Each depicted point corresponds to the individual quantitative measures obtained for each WT (*n* = 5) and APPswe/PS1dE9 (*n* = 6) per genotype group; *∗* indicates *p* < 0.05. NTg: wild type; Tg: APPswe/PS1dE9.

The Western blot analysis of APP expression in the studied young, 3-month-old APPswe/PS1dE9 and WT mice indicated a significant (*t*_(9)_ = 3.142; *p* = 0.0119) increase in expression in APPswe/PS1dE9 mice, which had a very large Cohen's *d* effect size, 1.981 (Figure 1(a)). No A*β* could be detected at this age by using either CT20 or 6E10 antibodies (Western blot) or ELISA (data not shown). Moreover, at this age, the *β*-C-terminal fragment (*β*CTF), the rate-limiting precursor to A*β*, was also virtually undetectable in the hippocampus of APPswe/PS1dE9 mice with either CT20 or 6E10 antibodies in Western blot assays (data not shown). These data suggest that the AD-like pathology in the studied 3-month-old APPswe/PS1dE9 mice may correspond to the presymptomatic stage of the human disease before clinical symptoms manifest [29].

We then asked whether, by analogy to our previous findings in TgCRND8 mice [23], APPswe/PS1dE9 mice display increased TNF*α* expression in the absence of detectable A*β*. Our data show that 3-month-old APPswe/PS1dE9 and WT mice express similar levels of TNF*α*, independent of the assay used for its detection (Figures 1(b) and 1(d), for Western blot and ELISA, resp.). However, the large Cohen's *d* effect size, 1.022, suggests that the difference in TNF*α* between groups may be meaningful. Interestingly, a linear regression analysis of the data from the APPswe/PS1dE9 mice indicated that the increase in APP correlates positively with TNF*α* (*r* = 0.9261; *p* = 0.008; Figure 1(c)), which resulted in a very large effect size: *r* = 0.93. Taken together, these data suggest that, despite the fact that the mean TNF*α* values between genotypes were similar, the APPswe/PS1dE9 mice expressing more APP also express more TNF*α*.

### 3.2. Induction of the Astrocyte Phenotypic Marker, GFAP, Points to Glial Activation in the Hippocampus of 3-Month-Old APPswe/PS1dE9 Mice

**Figure 2:**
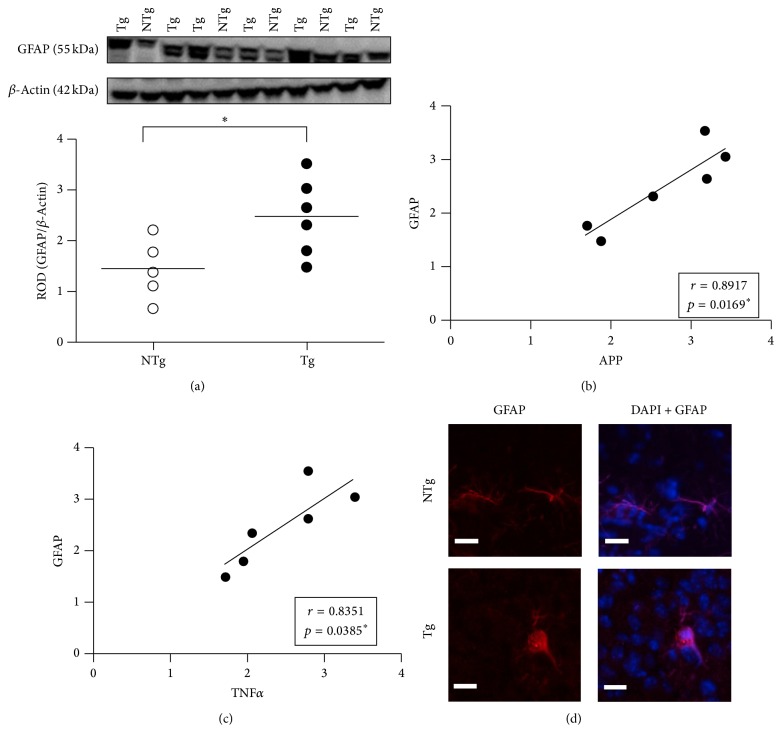
Quantification of GFAP expression in the hippocampus of 3-month-old WT and APPswe/PS1dE9 mice. (a) Representative immunoblot of GFAP (55 kDa) expression in hippocampal protein extracts coming from WT (*n* = 5) and APPswe/PS1dE9 (*n* = 6). Semiquantitative analysis of immunoblot was performed using ImageJ software. The immunoreactivity of each protein band was normalized to *β*-actin (42 kDa) immunoreactivity. The results were expressed as relative optical density (ROD); *∗* indicates *p* < 0.05. (b) The increase in GFAP correlates positively with APP (*r* = 0.8917; *p* = 0.0169) and (c) TNF*α* (*r* = 0.8351; *p* = 0.0385) among the APPswe/PS1dE9 mice analyzed according to the animal-by-animal basis. Linear regression analysis (b and c) was performed using the ROD values shown in Figures 2(a), 1(a), and 1(b) for GFAP, APP, and TNF*α*, respectively. *∗* indicates *p* < 0.05. (d) Confocal microscopy of the morphology of GFAP-IR cells (red) and DAPI for nuclei (blue) in the CA1-2 hippocampal subregion of WT (upper) and APPswe/PS1dE9 (lower) mice. Scale bar: 50 *µ*m. NTg: wild type; Tg: APPswe/PS1dE9.

The putative engagement of astrocytes along the activation pathway was explored by quantification of the GFAP phenotypic marker by Western blot. A significant increase in GFAP expression was found in APPswe/PS1dE9 mice as compared to WT (Figure 2(a); *t*_(9)_ = 2.485; *p* = 0.0347) with a very large Cohen's *d* effect size, 1.525. In the APPswe/PS1dE9 mice, this increase in GFAP is furthermore positively correlated with APP (*r* = 0.8917; *p* = 0.0169; Figure 2(b)). In addition, GFAP and TNF*α* expressions are also mutually correlated (*r* = 0.8351; *p* = 0.0385; Figure 2(c)). Both correlations yielded large effect sizes, *r* = 0.84 and 0.89, respectively.

Consistently, morphological analysis by confocal microscopy indicated that astrocytes display some signs of activation at this stage. Indeed, GFAP-IR cells with compact cell bodies and thick cellular processes were seen in the hippocampus and more specifically in CA1-2 subregion (Figure 2(d)). Collectively, these data suggest that the astrocytes from mice with higher APP overexpression are more advanced along the activation process (according to the higher GFAP expression) and produce more TNF*α*. These correlations may represent incremental changes at this presymptomatic stage of AD which progress towards recognized AD-like pathology.

### 3.3. The Levels of Hippocampal TSPO and Its Binding Site Do Not Differ between APPswe/PS1dE9 and WT Mice

**Figure 3:**
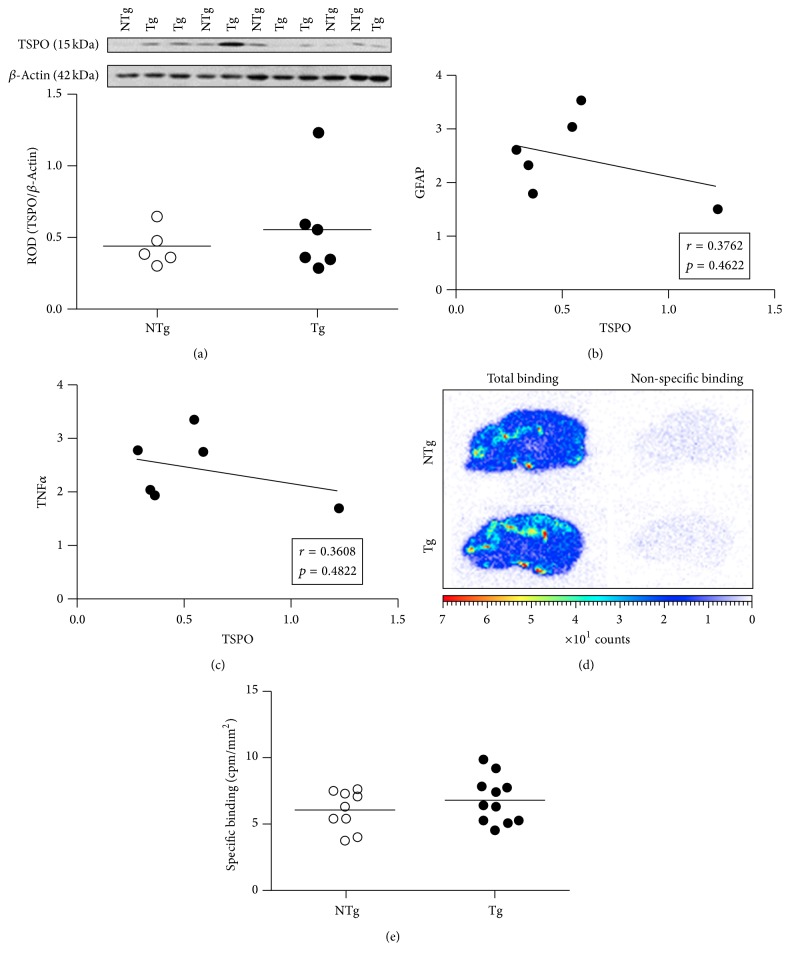
TSPO expression in the hippocampus of 3-month-old WT and APPswe/PS1dE9 mice. (a) Representative immunoblot of TSPO (15 kDa) in the hippocampus of WT (*n* = 5) and APPswe/PS1dE9 (*n* = 6) mice. Equal protein loading was confirmed by detection of *β*-actin (42 kDa) and results were expressed as relative optical density (ROD). (b) In APPswe/PS1dE9 mice, the correlations between TSPO and GFAP (*r* = 0.3762; *p* = 0.4622) and between TSPO and (c) TNF*α* (*r* = 0.3608; *p* = 0.4822) were not significant. Linear regression analysis (b and c) was performed using the ROD values shown in Figures 3(a), 2(a), and 1(b) for TSPO, GFAP, and TNF*α*, respectively. (d) Representative autoradiographic images obtained on 16 *µ*m thick sagittal brain sections: total (left panel) and nonspecific (right panel) binding in WT (upper) and T APPswe/PS1dE9 (lower) mice. (e) Quantitative autoradiographic measurements of TSPO density in the hippocampus were expressed as specific binding of [^3^H]PK-11195 in both groups. Data represent mean of cpm/mm^2^ for each WT (*n* = 9) and APPswe/PS1dE9 (*n* = 11) mouse. NTg: wild type; Tg: APPswe/PS1dE9.

TSPO induction has been proposed as a marker of full glia activation [30]. We therefore asked whether this marker is upregulated also during the very first stages of glial engagement along the activation process, as suggested by confocal microscopy (Figure 2(d)). A Western blot assessment of TSPO levels pointed to a heterogeneous expression in both APPswe/PS1dE9 and WT mice with a medium Cohen's *d* effect size, 0.467 (Figure 3(a)). However, no statistically significant difference was observed between the genotypes. The correlation between TSPO and GFAP expressions in APPswe/PS1dE9 group was not significant (*r* = 0.3762; *p* = 0.4622; Figure 3(b)) and TSPO induction was not correlated with TNF*α* (*r* = 0.3608; *p* = 0.4822; Figure 3(c)).

We reasoned that tissue homogenization in Western blot experiments might have masked a small TSPO induction and decided to further assess the in situ distribution of TSPO by autoradiography. The density of TSPO binding sites was therefore quantified on brain sections by [^3^H]PK-11195 autoradiography in the hippocampi of 3-month-old mice of both genotypes (Figures 3(d) and 3(e)). The PK-11195 added in excess (1 *μ*mol/L) almost completely inhibited the radioligand (1 nmol/L) binding. Globally, the total [^3^H]PK-11195 binding was relatively low all over the brain, although the hippocampus and periventricular region displayed an increased density of [^3^H]PK-11195 binding. No significant difference was observed between WT and APPswe/PS1dE9 groups (6.04 ± 0.49 versus 6.75 ± 0.52 cpm/mm^2^, resp.), in line with Western blot results. Altogether, the combined biochemical and autoradiographic evidence suggests that astrocytes in the hippocampus of 3-month-old APPswe/PS1dE9 mice have likely not yet reached a fully activated state (as attested by the absence of the TSPO marker induction). Nonetheless, according to the GFAP induction data and morphological alterations, they are clearly already engaged along the activation process.

### 3.4. Assessment of GFAP/TSPO Colocalization in the Hippocampus of APPswe/PS1dE9 versus WT Mice

**Figure 4:**
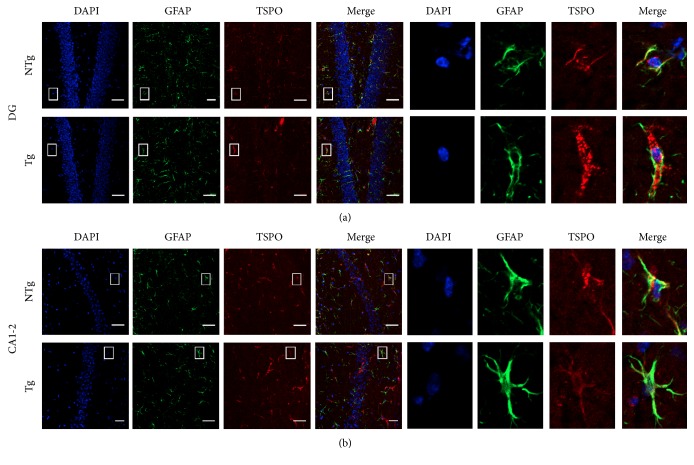
Immunofluorescence of GFAP/TSPO colocalization in hippocampal subregions of WT and APPswe/PS1dE9 mice at 3 months. Confocal staining of DAPI for nuclei (blue), GFAP for astrocytes (green), and TSPO (red) in DG (a) and CA1-2 (b) in WT (upper) and APPswe/PS1dE9 (lower) mice. A white square represents a magnified region of interest. Scale bar: 50 *µ*m.

**Figure 5:**
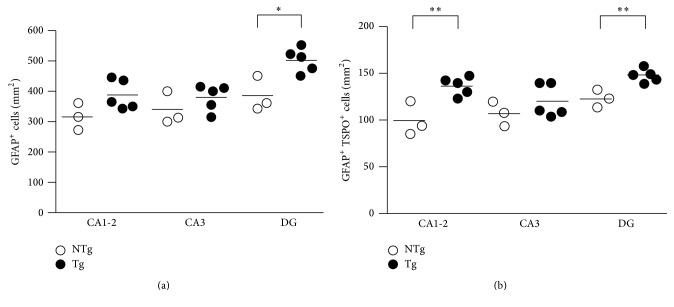
Semiquantitative assessment of GFAP/TSPO colocalization in hippocampal subregions of 3-month-old WT and APPswe/PS1dE9 mice. Quantification of (a) GFAP-IR cells and (b) GFAP/TSPO double-IR cells in CA1-2, CA3, and DG hippocampal subregions in WT (*n* = 3) and APPswe/PS1dE9 (*n* = 5) mice. Results were expressed as mean cell count per unit area (mm^2^) for each mouse; *∗* indicates *p* < 0.05 and *∗∗* indicates *p* < 0.01. NTg: wild type; Tg: APPswe/PS1dE9.

The relatively robust approaches, such as Western blot and autoradiography, allowed for the assessment of large regional changes. However, subtle differences may exist in hippocampal subregions important for learning and memory. Therefore, we performed a subregional immunohistochemical study on the hippocampal sections adjacent to those used for autoradiography (Figure 4). Analysis of GFAP-IR distribution as well as double GFAP/TSPO labeling pointed to a selective increase in the number of GFAP-IR cells (Figure 5(a)) in the DG (*t*_(6)_ = 3.495; *p* = 0.0129). The number of GFAP/TSPO double-IR cells (Figure 5(b)) was also apparently increased in the DG (*t*_(6)_ = 4.455; *p* = 0.0043) as well as in CA1-2 (*t*_(6)_ = 3.913; *p* = 0.0079) subregions of the hippocampus in APPswe/PS1dE9 mice (Figure 5(b)). This in situ analysis therefore indicated that, at a more resolute level, astrocytes also display upregulated GFAP expression which is detectable in the hippocampus of APPswe/PS1dE9 mice in a subregion-specific manner.

## 4. Discussion

The main finding of this study is that subtle, activation-related alterations are detectable in the hippocampus already during the presymptomatic stage of AD in the studied APPswe/PS1dE9 mouse model. These alterations include augmented expression levels of the astrocyte phenotypic marker, GFAP, as well as the hippocampal subregion-selective increase in the number of GFAP-IR cells. Combined with morphological signs (thickening of the cell bodies and processes) and the significant positive correlation existing between GFAP and TNF*α* expression, our data point to an altered state of astrocyte activation in the hippocampus of presymptomatic APPswe/PS1dE9 mice, which is compatible with their engagement along the activation pathway. Given the absence of the significant TSPO induction in biochemical (Western blot), autoradiographic ([^3^H]PK-11195 binding), and immunohistochemical (TSPO-IR) assays, the apparent increase in the number of double GFAP/TSPO-IR cells found in the DG and CA1-2 regions is likely due to the observed significant GFAP induction. As a corollary, the altered state of astrocyte activation, as reported here during the presymptomatic stage of AD, would differ from the astrocytes' fully activated state which is characterized by an upregulated TSPO expression [30].

The sole previous study that examined the relationship between early TNF*α* induction and APP/A*β* in APPswe/PS1dE9 mice used a bit older, 3.5-month-old animals, which display low, but detectable, soluble A*β* [31]. In order to more specifically assess the effect of APP overexpression in the absence of detectable amount of its cleavage products, we therefore used 3-month-old mice. In contrast to our study, the previous report did not specifically assess the hippocampus, yet it did show a significant increase in TNF*α* expression in the brain of 3.5-month-old APPswe/PS1dE9 mice [31]. Taken together, the previous study and our present data suggest that the presence of low A*β* may be sufficient and necessary to trigger a significant increase in TNF*α*. Besides, we have previously shown that 2-week difference in age during the presymptomatic stage is associated with the absence and the presence of AD-related alterations in hippocampal network synchronization, respectively [24]. We indeed found that, in another AD mouse model (TgCRND8), gamma-theta oscillation coupling is present (and similar to the one found in WT mice), whereas 2 weeks later (i.e., in 1-month-old mice), theta-gamma oscillation coupling is lost [24]. Moreover, in TgCRND8, in which the AD-like pathology develops much faster [32] than in APPswe/PS1dE9 [33] mice, a significant increase of TNF*α* level is detectable in the hippocampus already at 1 month of age [23]. In 3xTg-AD mice, increased TNF*α* has also been observed in the entorhinal cortex during the early stages of AD-like pathology, where it occurs along with monocyte chemoattractant protein-1 (MCP-1) induction [34]. The difference between our study and the two latter studies may be related to the use of different mouse models: APPswe/PS1dE9 (present study) versus TgCRND8 [23] and 3xTg-AD [34].

At this stage, the putative trigger of the TNF*α* in 3-month-old APPswe/PS1dE9 mice remains unknown. Indeed, neither A*β* nor *β*CTF, which have been previously suggested as possible TNF*α* inducers during the prodromal stage of amyloid pathology [23], were detectable in the present study. It remains to be established whether very low levels of these APP cleavage products may still be efficient in triggering elevated TNF*α* release. In agreement, low picomolar levels of A*β* (that could have remained undetectable by the Western blot and ELISA approaches used here) have been reported to positively impact synaptic transmission [35, 36]. Alternatively, the increase in APP above physiological levels, which is due to the transgene overexpression, may trigger TNF*α* induction. Accordingly, the capacity of APP to modulate synaptic plasticity of GABAergic neurons [37] may indeed act in synergy with TNF*α* to affect neuronal excitability as previously reported at this stage of AD pathogenesis in APPswe/PS1dE9 mouse [21]. Future studies, using the APP-overexpressing mice, are needed to test this interesting hypothesis.

In line with this hypothesis, our findings add to the evidence suggesting that there is a preinflammatory role of TNF*α* in the regulation of neuronal excitability during the very first stages of AD-like pathogenesis. Indeed, at the studied age (3 months), APPswe/PS1dE9 mice do not display a significant increase in A*β* or *β*CTF production (present study), yet they display hyperexcitability of hippocampal neurons [21]. According to this previous study, the increased excitability of hippocampal neurons translates to increased epileptiform activity, which manifests as nonconvulsive seizures and is associated with a decreased membrane potential [21]. Recent evidence from the hAPP-J20 mouse model of AD as well as in AD patients suggests that this hyperexcitability may be due to decreased GABAergic transmission resulting from decreased expression of the voltage-gated sodium channel subunit Nav1.1 [38] or GABAergic loss [39–42]. Furthermore, TNF*α* has been associated with synaptic dysfunction in AD such that TNF*α* antagonists were able to prevent A*β*-mediated inhibition of long-term potentiation (LTP) in vivo [43]. Alterations in excitability and synaptic plasticity, specifically in LTP, may underlie the cognitive deficits that are so typical of AD and pathological TNF*α* signaling may contribute to such impairments.

In contrast to the above-discussed early AD-associated glial alterations, which likely reflect increased cytokine levels, analogous changes at more advanced stages of pathology are widely recognized. For example, a previous study by Jimenez and colleagues using PS1 × APP mice detected a significant increase in hippocampal IL-1*β* and TNF*α* by 6 and 18 months of age, respectively [44]. Similar findings on the increase in expression of IL-1*β* and CXCL1 in 7-month-old and 9-month-old TgCRND8 mice, respectively, were also reported and were brain-region-specific [45]. A recent study from our group has also documented cytokine and TSPO induction in APPswe/PS1dE9 mice older than 6 months [46]. Interestingly, at these advanced stages of AD pathology, hyperexcitability, which may be related to at least TNF*α* and perhaps IL-1*β* at the early stages of pathogenesis [47], is not detectable anymore. The major challenge for the future is to understand the mechanisms underlying such a switch in cytokine actions in the course of AD progression, which may be related to the dual nature of TNF*α* signaling [48].

## 5. Conclusions

Our findings suggest that the mechanism of initial induction of TNF*α* may be related to overproduction of APP during the presymptomatic stage of AD. Indeed, increased APP triggers astrocyte activation in the absence of A*β*. Furthermore, the phenotypic marker of astrocyte activation (GFAP) is positively correlated with both APP and TNF*α*. Combined with the morphological signs of activation, these data indicate that astrocytes engaged into the activation process during the presymptomatic AD stage may be the source of TNF*α* even in the absence of full glial activation. Further studies aimed at discovering more sensitive biomarkers of astrocyte activation than TSPO and GFAP upregulation is now needed. They should help design new intervention strategies during the presymptomatic stages of the disease to provide a promising approach to halt the progression of AD.
